# Identification of Key Genes Affecting the Tumor Microenvironment and Prognosis of Triple-Negative Breast Cancer

**DOI:** 10.3389/fonc.2021.746058

**Published:** 2021-10-21

**Authors:** Jiarong Yi, Wenjing Zhong, Haoming Wu, Jikun Feng, Xiazi Zouxu, Xinjian Huang, Siqi Li, Zeyu Shuang

**Affiliations:** Department of Breast Oncology, Sun Yat-sen University Cancer Center, The State Key Laboratory of Oncology in South China, Collaborative Innovation Center for Cancer Medicine, Guangzhou, China

**Keywords:** TNBC, TCGA, GEO, estimate, tumor microenvironment

## Abstract

Although the tumor microenvironment (TME) plays an important role in the development of many cancers, its roles in breast cancer, especially triple-negative breast cancer (TNBC), are not well studied. This study aimed to identify genes related to the TME and prognosis of TNBC. Firstly, we identified differentially expressed genes (DEG) in the TME of TNBC, using Expression data (ESTIMATE) datasets obtained from the Cancer Genome Atlas (TCGA) and Estimation of Stromal and Immune cells in Malignant Tumor tissues. Next, survival analysis was performed to analyze the relationship between TME and prognosis of TNBC, as well as determine DEGs. Genes showing significant differences were scored as alternative genes. A protein-protein interaction (PPI) network was constructed and functional enrichment analysis conducted using the DEG. Proteins with a degree greater than 5 and 10 in the PPI network correspond with hub genes and key genes, respectively. Finally, CCR2 and CCR5 were identified as key genes in TME and prognosis of TNBC. Finally, these results were verified using Gene Expression Omnibus (GEO) datasets and immunohistochemistry of TNBC patients. In conclusion, CCR2 and CCR5 are key genes in the TME and prognosis of TNBC with the potential of prognostic biomarkers in TNBC.

## Introduction

Breast cancer is the second leading cause of cancer related deaths among women worldwide, with a prevalence of 11.7% and a mortality rate of 6.9% (1). The burden of breast cancer has grown in both developed and developing countries over time (2). In 2017 alone, it was estimated that 30% of all new cancer cases (252,710), among women in America, were breast cancer (3). Based on the characteristics of molecular markers, breast cancer is divided into 3 major subtypes, namely estrogen receptor positive and progesterone receptor positive (luminal A, luminal B), HER2 overexpression (HER2+) and triple negative breast cancer (TNBC). Triple negative breast cancer (TNBC) is the subtype of breast cancer that tests negative for estrogen receptors (ER), progesterone receptors (PR), and excess HER2. Worldwide, TNBC accounts for about 15% of the total breast cancer cases (4), and 83% of disproportionate deaths compared to other breast cancer subtypes (5). The growth of TNBC is not triggered by the HER2 protein or the hormones estrogen and progesterone. Therefore, the cancer does not respond to targeted therapy with HER-2 receptor, monoclonal antibody and endocrine therapy. Although various treatments and medicines used to manage TNBC are constantly developing, more than 70% of patients have recurrence and relapse within 3 years after surgical resection with poor prognosis (6). Also, standardized TNBC treatment regimens are still lacking (7). Researchers are trying to find out whether certain medications can interfere with the processes that cause TNBC to grow (8). Therapeutic approaches that target the TME have been suggested as promising strategies in cancer treatment.

The tumor microenvironment (TME) is the cellular and immune environment surrounding the primary tumor. There are many kinds of cells and molecules in TME, including immune cells, extracellular matrix proteins, blood vessels and cytokines. Tumor cells interact with molecules and cells in the TME. Recent literature shows that the immune landscape of the TME can promote or inhibit tumor initiation and progression (9–11). In fact, findings from clinical trials have revealed the potential of a number of therapeutic strategies targeting the TME for cancer therapy. However, little research has described the role of TME in the progression of triple negative breast cancer (TNBC). TNBC is characterized by a unique TME, which differs from other breast cancer subtypes. In TNBC patients, the TME is associated with induction of proliferation, angiogenesis, inhibition of apoptosis and immune system suppression, and drug resistance (12, 13). Nonetheless, the functional tumor infiltrating lymphocytes, the mechanism of TME regulation and concerning predictive biomarkers remain unclear (14–16).

The present study aimed to identify key genes associated with TNBC microenvironment and prognosis of patients. Summarily, the relationship between the key genes and prognosis of TNBC patients was analyzed, based on datasets from the Cancer Genome Atlas (TCGA) database and Gene Expression Omnibus (GEO) databases, which comprise gene expression and quantification data as well as clinical information of TNBC patients. Estimation of Stromal and Immune cells in Malignant Tumor tissues using Expression data (ESTIMATE) website provides easy access to predicting infiltration of immune cells and stromal cells in TME, while CIBERSORT provide 22 immune cell information and 547 immune-related markers in TNBC. Based on this information, we screened out key genes in the TNBC microenvironment and elucidated their association with prognosis of TNBC patients.

## Materials and Methods

### Study Design

**Figure 1:**
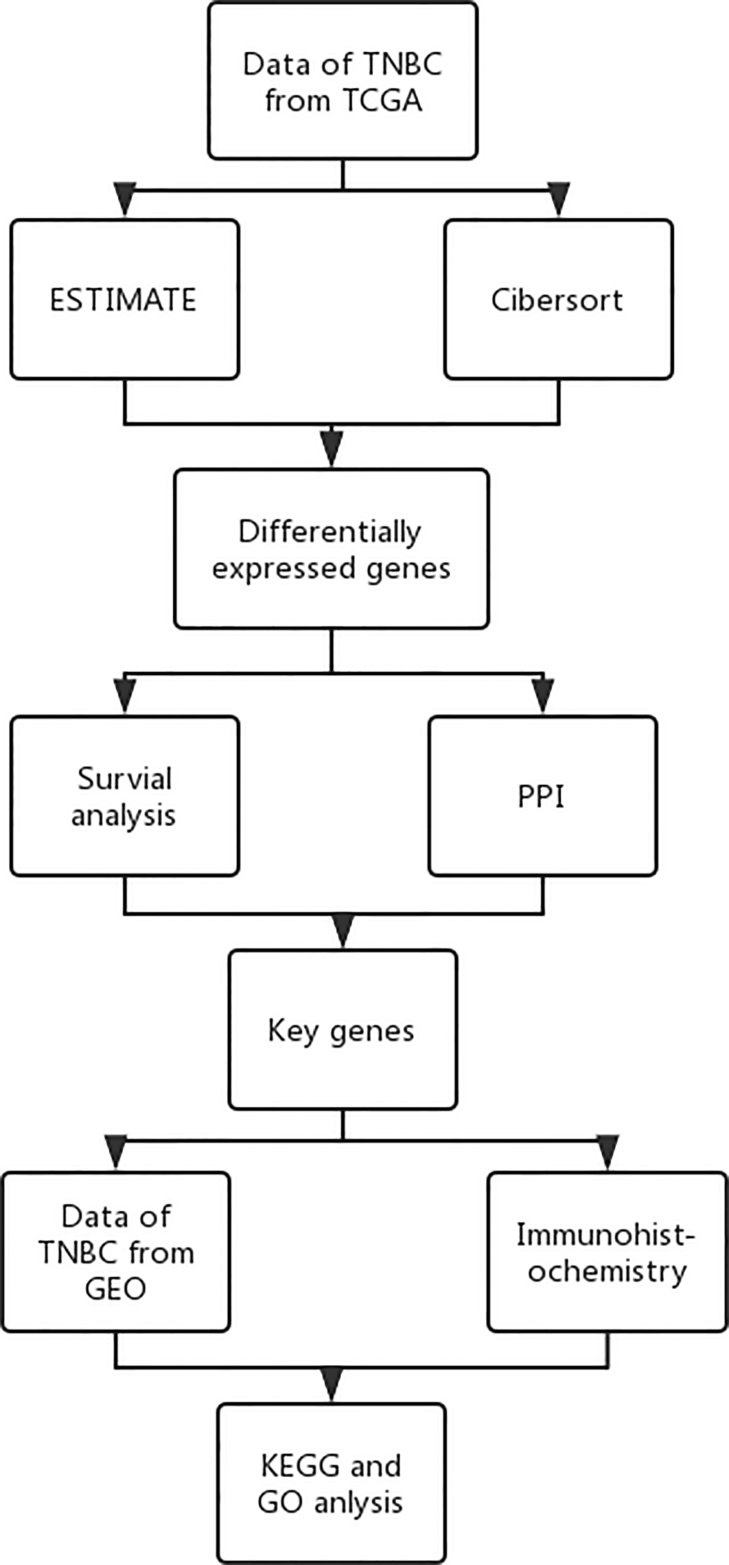
Research route.

A schematic representation of the whole research is presented in **Figure 1**.

### Gene Expression Dataset

Triple negative breast cancer (TNBC) datasets were obtained from the TCGA and GEO databases. The TCGA dataset comprised basic information, gene expression profiles and prognostic information. This study only included patients who had been diagnosed with TNBC with clear pathology and clinical information, with those who had insufficient or missing data such as age, TNM staging, and OS excluded. Data from GEO was searched using “TNBC” and “survival”, as key terms, using similar inclusion criteria applied in TCGA. Finally, information belonging to 116 patients was obtained. The final screening outcome was the GSE31519 dataset, which comprised information for 68 patients. And the patient characteristics were showed in **Table 1**.

**Table 1:** Patient characteristics (n = 184 patients).

Variables		No. Patients (%)
**Gender**		
	Female	184 (100.0%)
	Male	0 (0.0%)
**Age (y)**		
	65 and less	30 (16.3%)
	65 and above	154 (83.7)
**Tumor size**		
	T1	38 (20.7%)
	T2	112 (60.9%)
	T3	22 (11.9%)
	T4	12 (6.5%)
**Nodal status**		
	N0	103 (56.0%)
	N1	45 (24.5%)
	N2	25 (13.6%)
	N3	11 (5.9%)
**Metastasis**		
	M0	160 (87.0%)
	M1	8 (4.3%)
	Mx	16 (8.7%)
**Stage**		
	I	29 (15.8%)
	II	103 (55.9%)
	III	44 (24.0%)
	IV	8 (4.3%)

### Analysis of Immune Infiltration in the TME

Relative proportions of infiltrating immune cells for TNBC were analyzed using ESTIMATE and CIBERSORT (https://cibersort.stanford.edu/). Briefly, the ESTIMATE score was analyzed with the R package, by comparing all patients’ information with the standard information from the R package, then scoring their stromal and immune scores (17, 18). The CIBERSORT score was analyzed using the R package, then the relative proportion of 22 types of infiltrating immune cells used to determine 547 immune-related markers in TNBC patients. The R package of CIBERSORT quantifies the relative scores of immune cells and analyzes the relative proportion of immune cells (19, 20).

### Identification of Differentially Expressed Genes

TNBC samples were assigned to high and low immune score groups, based on the median immune score obtained in ESTIMATE analysis. On the other hand, the TNBC samples were assigned into high stromal and low stromal score groups, based on the median stromal score obtained from ESTIMATE analysis. Thereafter, differentially expressed genes (DEGs) were identified across different groups using the limma package in R (21). The threshold of differentially expressed genes were: |log2 fold change (log2FC)| > 1 and false discovery rate (FDR) < 0.05. All results were presented using a heat map.

### Identification of Key Genes in the TME

Alternative genes associated with both the immune and stromal scores were screened, and results presented using a Venn diagram. Next, the proteins corresponding with alternative genes were used to construct a PPI network *via* the STRING database (22), with the degree of proteins indicating the number of edges linking a given node protein. Protein to gene interactions with integrated and scores > 0.95 selected, while proteins with proportions greater than 5 and 10 selected as hub and key proteins, corresponding with hub and key genes.

### Further Verification of the Key Genes

Key genes were verified based on the GEO database and *via* immunohistochemistry. Briefly, data from both the TCGA and GEO databases were subjected to ESTIMATE and survival analyses. Next, immunohistochemistry was carried out on 26 samples collected from TNBC patient at the Cancer Center of Sun Yat-sen University, and the basic information of patients were showed in **Table 2**. Summarily, the tissues were first dewaxed in xylene, rehydrated in alcohol, and blocked in endogenous peroxidase activity, then incubated overnight at 4°C with specific antibodies targeting CCR2 (rabbit; 1:100, Abcam, Cambridge, UK) or CCR5 (rabbit; 1:500, Abcam). The samples were then incubated at room temperature with secondary antibodies (ab97080, goat anti-rabbit, 1:2,000; ab97040, goat anti-mouse, 1:500, Abcam) for 10 min, and in 3-3’-diamino-benzidine for 1.5 min. Thereafter, the samples were counter stained with hematoxylin for 30s and visualized under a microscope. Based on the degree of staining, the samples were divided into either high (CCR5+ or CCR2+) or low (CCR5-or CCR2-) expression groups, using the imagine gray scale. The resulting clinical information was used to perform survival analysis using the survival and survminer packages in R (23).

**Table 2:** Basic information of patients used in immunohistochemistry.

Variables		No. Patients (%)
**Gender**		
	Female	26 (100.0%)
	Male	0 (0.0%)
**Age (y)**		
	65 and less	22 (84.6%)
	65 and above	4 (15.4%)
**Tumor size**		
	T1	2 (7.6%)
	T2	18 (69.2%)
	T3	2 (7.6%)
	T4	4 (15.3%)
**Nodal status**		
	N0	12 (46.2%)
	N1	8 (30.8%)
	N2	3 (11.5%)
	N3	3 (11.5%)
**Metastasis**		
	M0	24 (92.2%)
	M1	1 (3.9%)
	Mx	1 (3.9%)
**Stage**		
	I	2 (7.6%)
	II	13 (50.0%)
	III	10 (38.5%)
	IV	8 (3.9%)

### Analysis of Potential Mechanism Through Which Key Genes Influence the TME

Expression across different immune infiltration groups of TNBC patients was subjected to GO and KEGG analysis with the aim of elucidating the potential mechanism through which key genes influence the TME. GO and KEGG analyses were performed using the clusterProfiler, DOSE, and enrichplot packages in R (24, 25). To further analyze activity of key genes in different groups, GSEA was carried out where necessary depending on the potential signal pathways (26).

## Results

### Relationship Between ESTIMATE Score and Prognosis of TNBC Patients

**Figure 2:**
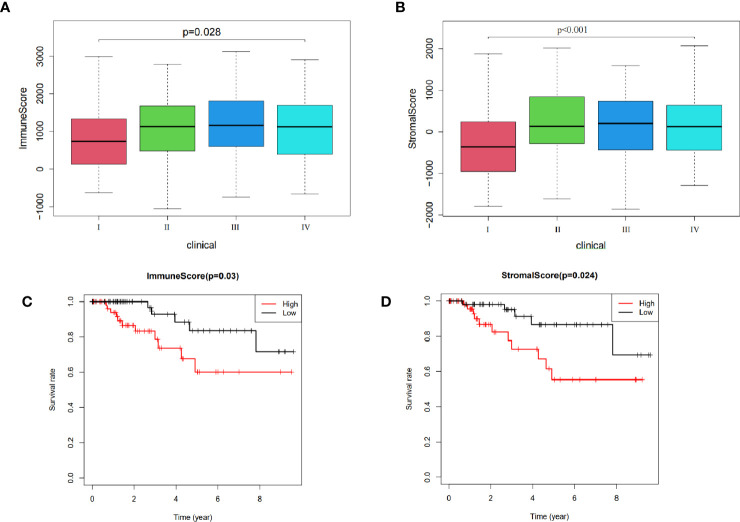
Relationship between ESTIMATE scores and prognosis of TNBC patients: **(A)** The influence on stage of immune score. **(B)** Effect on stage of stromal score. **(C)** Effect on prognosis of immune score. **(D)** Effect on prognosis of stromal score.

Results from ESTIMATE analysis revealed several score groups, namely high and low immune, and high stromal and low stromal score groups. Results from survival analysis across all groups showed that immune and stromal scores significantly influenced the TMN stage of TNBC patients at p=0.028 and p < 0.001, respectively (**Figures 2A, B**). Patients at stage IV exhibited significantly higher immune and stromal scores than those at stage I. Moreover, immune score (p=0.03) and stromal score (p=0.024) significantly influenced prognosis of TNBC patients (**Figures 2C, D**). Overall, these results indicated that low immune and stromal scores are indicators of better prognosis of TNBC patients.

### Identification of Differentially Expressed Genes

**Figure 3:**
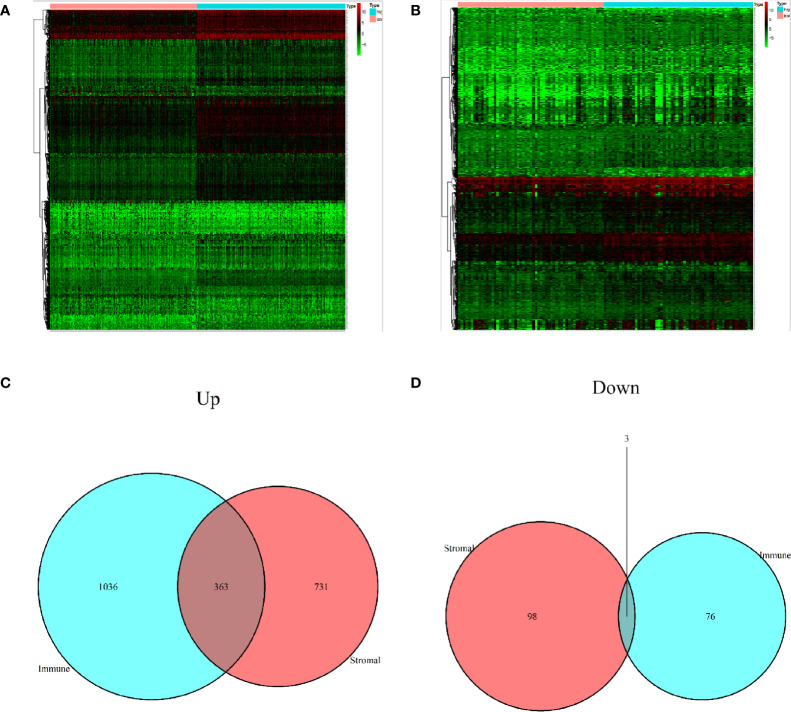
Identification of differentially expressed genes. **(A)** A heat map showing differentially expressed genes in the immune score. **(B)** A heat map showing differentially expressed genes in stromal score. **(C)** Upregulated DEGs. **(D)** Downregulated DEGs.

A comparison between high with low immune score group, as well as high with low stromal score group, revealed a total of 2307 DEGs. Among them, 2130 and 177 were up-regulated and down-regulated, respectively, of which 363 in the upregulated and 3 in the downregulated groups exhibited potential to influence both immune and stromal scores (**Figures 3A–D**).

### Identification of Key Genes in TME

**Figure 4:**
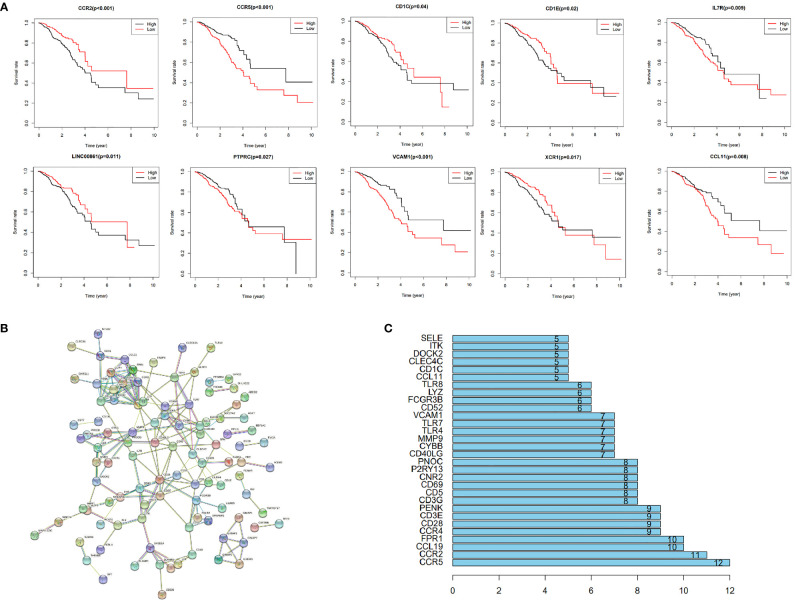
Among the 366 genes that were screened out, survival analysis for every gene resulted in 10 significant (p < 0.05) genes, namely CCR2, CCR5, CD1C, CD1E, IL7R, LINC00861, PTPRC, VCAM1, XCR1 and CCL11 **(A)**. These were regarded alternative genes and were used for identification of key genes in the TME. A PPI network, constructed *via* the STRING database, showed important node genes, with the degree of genes indicating the number of edges linking to a given node gene **(B)**. The degree of interaction for each gene was calculated and hub genes with values greater than5 and 10, including CCR2 and CCR5 selected **(C)**.

Among the 366 genes that were screened out, survival analysis for every gene resulted in 10 significant (p<0.05) genes, namely CCR2, CCR5, CD1C, CD1E, IL7R, LINC00861, PTPRC, VCAM1, XCR1 and CCL11 (**Figure 4A**). These were regarded alternative genes and were used for identification of key genes in the TME. A PPI network, constructed *via* the STRING database, showed important node proteins indicating node genes, with the degree of proteins indicating the number of edges linking to a given node proteins (**Figure 4B**). The degree of interaction for each protein was calculated and hub proteins with values greater than5 and 10, including CCR2 and CCR5 selected (**Figure 4C**). All hub proteins correspond with hub genes.

### Verification of the Key Genes

**Figure 5:**
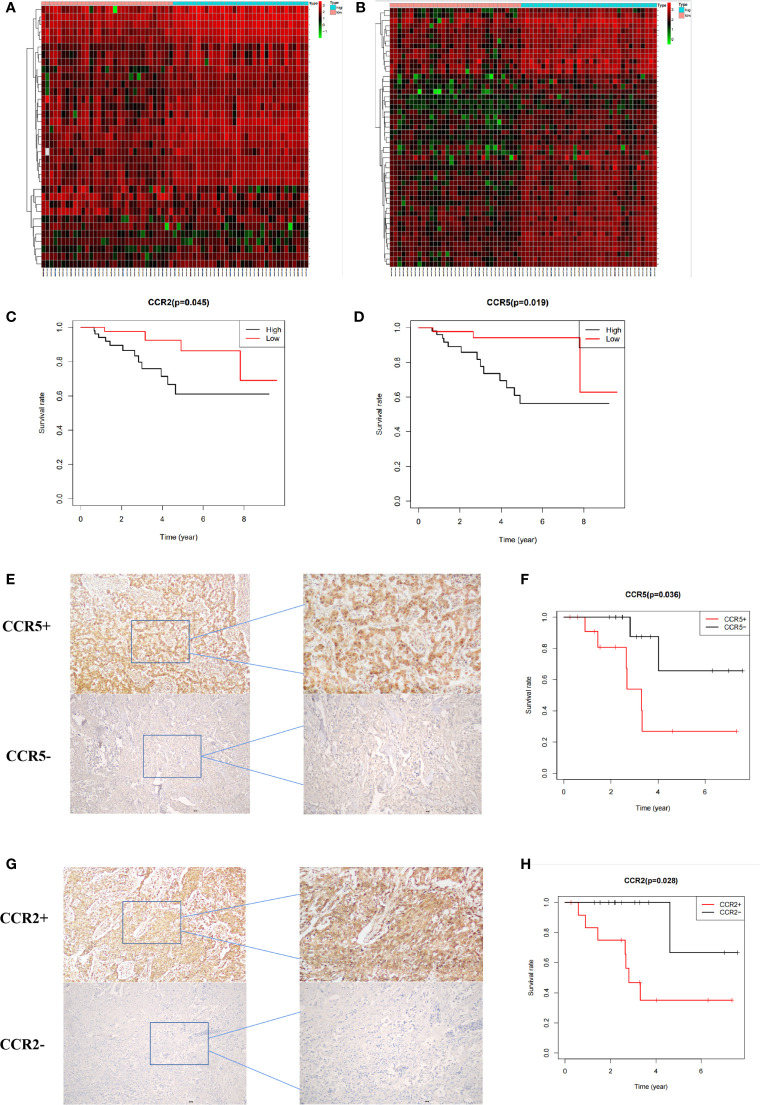
Verification of key genes. **(A, B)** Heat maps showing differentially expressed genes in patients with different immune and stromal scores. **(C, D)** Prognosis of patients with different expression of CCR2 and CCR5 in the GEO database. **(E F)** Prognosis of 26 patients with different CCR5 expression. **(G, H)** Prognosis of patients with different CCR2 expression.

Data obtained from the GEO database and immunohistochemistry were analyzed for verification of the key genes. DEGs with different immune and stromal scores are shown in **Figures 5A, B**. Results from survival analysis showed that upregulation of CCR2 and CCR5 was associated with poor prognosis of TNBC patients (**Figure 5C, D**). Results from immunohistochemistry and survival analysis further revealed poor prognosis of patients with high expression CCR2 (**Figures 5E, F**) and CCR5 (**Figures 5G, H**). Overall, these results confirmed that CCR2 and CCR5 are key genes in the TME of TNBC.

### Potential Mechanism of Action

**Figure 6:**
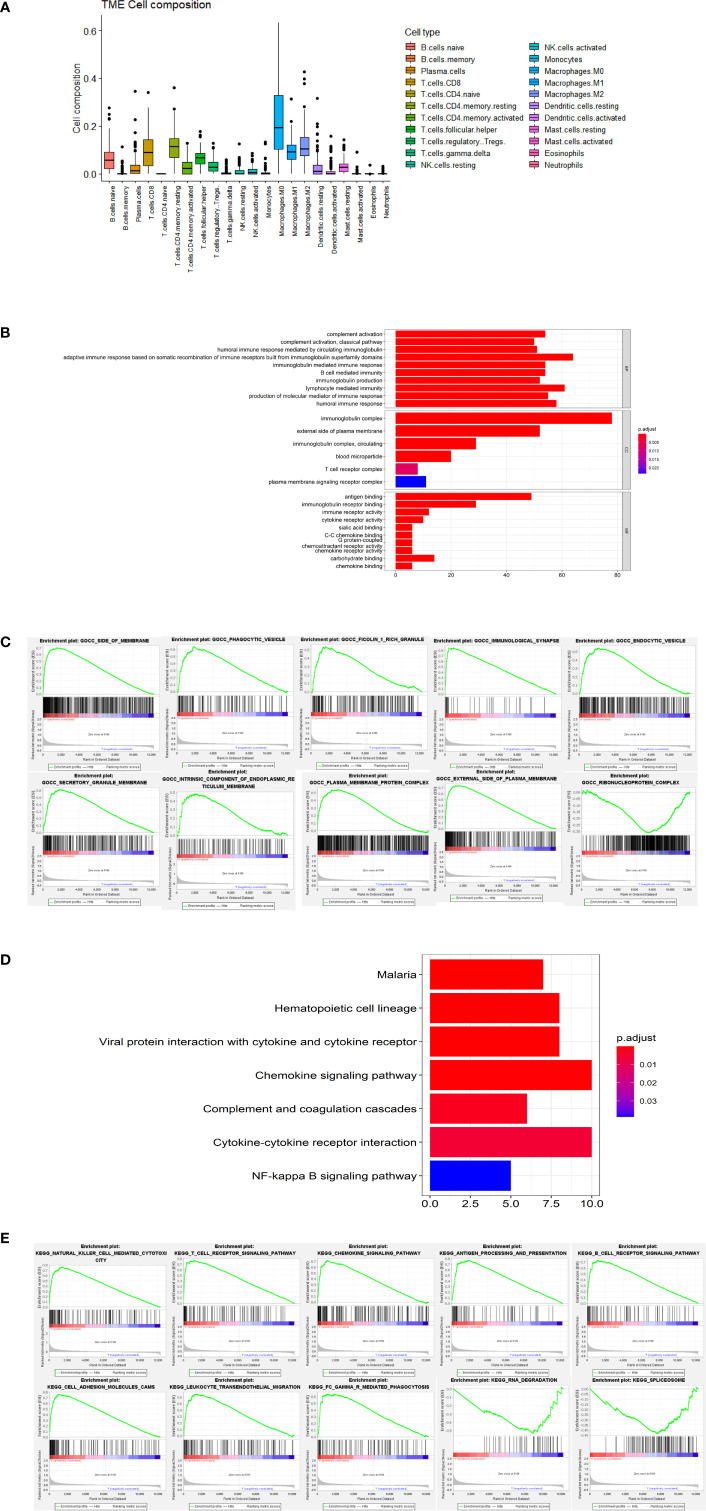
Potential mechanism of action. **(A)** Results from CIBERSORT showing infiltration of 22 immune cells. **(B)** Results of GO analysis showing the main significantly enriched GO terms. **(C)** The top 10 enriched GO terms in GSEA. **(D)** Results of KEGG analysis showing the main significantly enriched pathways. **(E)** The top 10 signaling pathways in GSEA.

Results from CIBERSORT analysis for immune cell infiltration, and macrophages revealed that M0 was the main infiltrating cell (**Figure 6A**). In addition, results from GO functional enrichment and KEGG pathway enrichment analysis as well as GSEA revealed top 10 enriched GO terms, including SIDE OF MEMBRANE and PHAGOCYTIC_VESICLE (**Figures 6B, C**). The top 10 signaling pathways, including NATURAL KILLER CELL MEDIATED CYTOTOXICITY and T CELL RECEPTOR SIGNALING PATHWAY, are shown in **Figures 6D, E**. These results indicate the potential mechanism of CCR2 and CCR5 influencing TME in TNBC.

## Discussion

Triple negative breast cancer (TNBC) is a largely hostile subtype of breast cancer, with a high possibility of metastasis and a lack of explicit targets for targeted therapeutics. In fact, TNBC is considered to have an exceptional TME, that is different from other subtypes (13). Previous studies have shown that the interactions between cancer cells and components of the TME play significant roles in cancer proliferation and metastasis (27, 28). Therefore, identification of key genes associated with TNBC’s TME is critical for development of effective management and treatment strategies for TNBC. In the present study, we identified 366 differentially expressed genes influencing both immune scores and stromal scores. Among them, 30 were hub genes in PPI network, of which CCR2 and CCR5 were identified as the key genes that influence TME and prognosis of TNBC patients. These were further verified *via* immunohistochemistry and data from the GEO database.

The CCL2-CCR2 signaling axis plays multiple pro-tumorigenic roles, such as mediating tumor growth and angiogenesis as well as usurping host stromal cells to support tumor progression (29). Previous studies have also demonstrated the translational potential of CCL2/CCR2 in hepatocellular carcinoma, pancreatic cancer and esophageal squamous cell carcinoma (30–32). Tumor development may be favored by the CCL5-CCR5 signaling axis favor in multiple ways, including proliferation, immunosuppression, angiogenesis, and migration (33–35). Most studies on CCL5-CCR5 have focused on gastric cancer and pancreatic cancer (36, 37). and found that both signaling axes are closely related to immune cells, where they augment their functions, induce their differentiation and promote their migration to TME. Results of the present study are consistent with findings from previous studies which indicated that high expression of CCR2 and CCR5 promotes tumor progression (38, 39). In fact, CCL2-CCR2 and CCL5-CCR5 signaling axes promote migration of cancer cells in breast cancer, thus are potential targets for development of breast cancer therapy. Therefore, identification of CCR2 and CCR5 as key genes in the TME of TNBC is expected to aid in future development of targeted therapies against the subtype.

The immune cells showed different infiltration in different ESTIMATE score groups, including macrophages, T cell and CD8^+^. These immune cells are significant components of TME and have various functions in cancer proliferation and metastasis (40). The main signaling pathways in different ESTIMATE score groups were related to immunomodulation, and included NATURAL KILLER CELL MEDIATED CYTOTOXICITY and T CELL RECEPTOR SIGNALING PATHWAY. The observed changes in immune cell infiltration and activation of signaling pathways may be related to differential expression of CCR2 and CCR5, although the actual underlying mechanism needs further exploration.

## Conclusion

In summary, CCR2 and CCR5 are key genes influencing the TME of TNBC, and have significant effects on prognosis of TNBC patients. Both genes have potential predictive ability, hence can be used as biomarkers in targeted development of therapies for treatment of TNBC. In future, unraveling the mechanism underlying these hallmarks of TNBC will be key in ensuring their clinical application for TNBC treatment.

## Data Availability Statement

The datasets presented in this study can be found in online repositories. The names of the repository/repositories and accession number(s) can be found in the article/supplementary material.

## Author Contributions

JY and ZS designed the study. JY and WZ wrote the manuscript. HW, SL, and XZ revised and polished the manuscript. JY, HW, JF, and XH performed the experiments and statistical analysis of the data. All authors contributed to the article and approved the submitted version.

## Funding

This work was supported by project grants from the National Natural Science Foundation of China (81802663).

## Conflict of Interest

The authors declare that the research was conducted in the absence of any commercial or financial relationships that could be construed as a potential conflict of interest.

## Publisher’s Note

All claims expressed in this article are solely those of the authors and do not necessarily represent those of their affiliated organizations, or those of the publisher, the editors and the reviewers. Any product that may be evaluated in this article, or claim that may be made by its manufacturer, is not guaranteed or endorsed by the publisher.
